# Investigating Recent Testing among MSM: Results from Community-Based HIV Rapid Testing Attendees in France

**DOI:** 10.1155/2013/648791

**Published:** 2013-07-25

**Authors:** Nicolas Lorente, Karen Champenois, Jérôme Blanche, Marie Préau, Marie Suzan-Monti, Marion Mora, Lionel Fugon, Maria Patrizia Carrieri, Luis Sagaon-Teyssier, Jean-Marie Le Gall, Bruno Spire, Yazdan Yazdanpanah

**Affiliations:** ^1^INSERM, UMR912 (SESSTIM), 13006 Marseille, France; ^2^Aix Marseille Université, UMR_S912, IRD, 13006 Marseille, France; ^3^ORS PACA, Observatoire Régional de la Santé Provence Alpes Côte d'Azur, 13006 Marseille, France; ^4^ATIP/AVENIR, INSERM U738, 75877 Paris, France; ^5^GREPS, Université Lyon 2, 69500 Bron, France; ^6^AIDES, 93508 Pantin, France; ^7^Coalition PLUS, 93508 Pantin, France; ^8^Service des Maladies Infectieuses et Tropicales, Hôpital Bichat-Claude Bernard, 75877 Paris, France; ^9^Université Denis Diderot, 75877 Paris, France

## Abstract

*Background*. We aimed to identify factors associated with recent HIV testing in MSM who attended two experimental community-based and nonmedicalized voluntary counselling and testing programmes (CB-VCT) targeting MSM in France. *Methods*. This analysis was based on data collected in 2009–2011 through a self-administered pretesting questionnaire. An index measuring the level of participants' sexual orientation disclosure was built: the higher the index, the greater the disclosure. Factors associated with recent HIV testing (last test ≤ 1 year) were identified using a multivariate logistic regression model adjusted for the CB-VCT programme of enrolment. *Results*. 716 MSM provided data on testing history. Overall, 49% were recently tested for HIV and 51% were not. Recently tested MSM had a higher homosexuality disclosure index (adjusted OR [95% confidence interval]: aOR = 1.2 [1.1–1.4]), reported more inconsistent condom use during anal sex with men (aOR = 1.6 [1.2–2.1]), and were less likely to have sex under the influence of club drugs (aOR = 0.6 [0.4–1.0]). *Conclusion*. New testing strategies should focus on those who live their homosexuality relatively secretly and those who use club drugs before sex. Governments should develop policies which encourage improved social acceptance of homosexuality as concealment of sexual orientation represents a major barrier to testing.

## 1. Introduction

In resource-rich countries, men who have sex with men (MSM) are greatly affected by the HIV burden [1–7], including France where they account for 40% of the annual new diagnoses [8]. The French HIV incidence in MSM is 60 times higher than that in the overall population [2]. Although a large proportion of MSM have already been tested for HIV in France [7, 9, 10], it is estimated that they account for 31% of the hidden epidemic [11] and for 19% of the diagnoses made at an advanced disease stage in 2011 (CD4 < 200/mm^3^, [8]). 

HIV testing has now become a tool to limit the HIV epidemic [12] and is a recognized element of combination prevention based on biomedical (preexposure prophylaxis, treatment as prevention) and behavioural (mainly serosorting and positioning) tools [13–17]. Indeed, knowledge of HIV serostatus is the cornerstone of successful combination prevention, as the latter's implementation is adapted according to the individual's serological status.

In France, just as in the USA, guidelines encourage the extension of HIV testing and recommend annual testing of certain population groups at high risk of acquiring HIV, in particular MSM [18–20]. Early detection of HIV leads to adequate linkage to care and treatment initiation, which in turn reduce viral load and limit onward transmission [13, 14, 21]. Furthermore, it has been demonstrated that half of new HIV contaminations are due to people who are unaware of their HIV infection [22]. 

Barriers to HIV testing have been highlighted in MSM as well as in the general population. These include the individual's perception of low or no risk of being infected, the fear of testing positive, and concerns about confidentiality and structural barriers, such as the time needed to take the test [23–25]. In addition, inappropriate counselling and moralistic attitudes regarding MSM sexual practices and regarding their testing habits were reported by the gay community as reasons for not testing [26]. 

The first step taken to overcome such barriers was to bring new HIV testing offers to MSM living in France through two community-based and nonmedicalized voluntary counselling and testing (CB-VCT) programmes: ANRS-COM'TEST [27] and ANRS-DRAG [28]. Testing was included in a comprehensive strategy of HIV exposure risk reduction where sexuality was openly addressed with peers. Among MSM who participated in the ANRS-COM'TEST, roughly 30% had not been tested for at least two years and reported HIV at-risk behaviours [27]. In order to increase repeat testing, the next step was to understand what leads MSM to go for testing or not. This study aimed to identify factors associated with recent testing in MSM who attended the two CB-VCT programmes (ANRS-COM'TEST and ANRS-DRAG) in France.

## 2. Methods

### 2.1. Intervention and Population

This analysis is based on data from two French CB-VCT programmes using rapid HIV tests exclusively targeting MSM: ANRS-COM'TEST and ANRS-DRAG, reported in detail elsewhere [27, 28]. 

In brief, both studies were cross-sectional and assessed a nonmedicalized voluntary counselling and testing offer implemented by community members from the French NGO *AIDES*, a community-based organization that focuses on outreach and prevention services for HIV-exposed populations, including MSM. Although they are not professional healthcare workers, *AIDES* staff members performed the entire testing procedure using HIV rapid tests and provided specific counselling based on the motivational interview method which they were trained in [29]. The studies were carried out during dedicated weekend or evening sessions once or twice a week. MSM were informed about the availability of the CB-VCT through communication campaigns in gay venues and on the Internet (posters, flyers, web banners, and ads). Eligibility criteria were as follows: being older than 18, being a man, and reporting to have sex with other men.

The ANRS-COM'TEST study was conducted from February 2009 to June 2010 in four French cities: Paris, Lille, Montpellier, and Bordeaux. The study was implemented at various *AIDES* premises, where potential participants came to be tested following the communication campaign [27]. The ANRS-DRAG study was conducted from March 2010 to April 2011 in free and anonymous VCT centres based in three French cities (Paris, Marseille, and Nice), outside of opening hours. The centres were only open during these hours to MSM who came to be tested using the CB-VCT offer and not to their usual attendees [28].

Both studies were approved by the French comité de protection des personnes (ANRS-COM'TEST: Nord-Ouest III, ANRS-DRAG: Sud-Est III) and the Agence française de sécurité sanitaire des produits de santé (AFSSAPS). All participants had to provide written informed consent before enrolment, and the studies were anonymous.

### 2.2. Data Collection

All participants had to fill in self-administered questionnaires during the testing procedure. The present analysis was based on data from the pre-testing questionnaire that collected sociodemographic characteristics, risk perceptions, HIV testing history, and sexual behaviour in the previous six months. Similar questionnaires were used in both studies, allowing us to merge databases.

### 2.3. Main Outcome and Explanatory Variables

Participants were asked when their last HIV test had been. This variable was then dichotomised into “recently tested” (i.e., the most recent HIV test performed in the 12 months prior to the CB-VCT) versus “not recently tested” (i.e., the most recent HIV test performed more than 12 months prior to the CB-VCT or having never been tested). This 12-month cutoff was chosen in accordance with French testing guidelines which recommend a test every year for MSM [18]. 

Several potential explanatory variables were computed using one or more questions. We built an index of sexual orientation disclosure, measuring the level of disclosure of one's sexual orientation with the question: “Is your homo-, bisexuality known to your …” and broken down for each of the following categories: father, mother, brother(s) or sister(s), colleagues, and heterosexual friends. The higher the index, the greater the participant's disclosure of his sexual orientation. 

Participants were asked to answer several questions about anal sex and condom use with their casual and/or steady partners. We then computed a risk proxy variable of inconsistent condom use (ICU): participants who reported that they had not systematically used condoms during anal intercourse in the previous six months, irrespective of the type of partner, were classified as having ICU.

The questionnaire also collected information about sex under the influence of psychoactive products. The following potential explanatory variables were therefore considered: alcohol, poppers, cannabis, and club drugs grouped together (ecstasy, MDMA, cocaine, and crack).

### 2.4. Statistical Analysis

A logistic regression model was built to determine factors associated with the outcome, that is, with the fact of having been tested in the last 12 months. This model was adjusted for the specific study in which participants were enrolled (ANRS-COM'TEST or ANRS-DRAG) in order to take into account the possible recruitment biases in both studies. Potential explanatory variables of recent testing were individually screened in univariate analyses. Variables achieving a significance level of ≤0.25 were considered eligible for inclusion in the multivariate model. A backward method based on the log-likelihood ratio test (entry threshold *P*-value ≤ 0.05) was then used to select factors independently associated with the outcome. A sensitivity analysis was performed: two additional multivariate models were built using different cut-off points for the outcome variable (11 and 13 months versus 12 in the base-case analysis). 

Statistical analyses were performed using SPSS-17 software (SPSS, Inc., Chicago, Illinois, USA) and STATA 12 (StataCorp. 2011. Stata Statistical Software: Release 12. College Station, TX: StataCorp LP).

## 3. Results

### 3.1. Study Population

Overall, 743 MSM participated in the ANRS-COM'TEST and the ANRS-DRAG studies. Complete data on the most recent HIV test were available for 716 MSM (our study group). The other 27 were excluded from this analysis: 5 men gave no information about history of HIV testing and 22 reported having been previously tested but did not specify the date of the most recent test. 

Among the 716 MSM included in this analysis, 517 (71%) and 199 (28%) were enrolled in the ANRS-COM'TEST and ANRS-DRAG studies, respectively. Median age was 31 (interquartile range, IQR = [25–39]); 81% defined themselves as homosexual. Regarding the main outcome, 349 MSM (49%) were recently tested, that is, reported at least one test in the 12 months previous to the study. Among the 367 MSM (51%) not recently tested, 146 (40%) had been tested more than one year but less than two years previously. For the 221 (60%) not tested within the previous two years, the median time since their last test was 46 months (IQR = [33–64]).

### 3.2. Comparison of MSM Regarding the Outcome

MSM characteristics according to recent and not recent HIV testing are shown in Table 1. Compared with their not recently tested counterparts, univariate analyses showed that MSM recently tested were younger (median age: 30 versus 32 years, *P* = 0.03), defined themselves more often as homosexual (86% versus 77%, *P* = 0.06), and were more often victims of verbal abuse because of their sexual orientation (24% versus 16%, *P* = 0.005). 

Interestingly, the MSM recently tested had disclosed their homosexuality more than those not tested recently with, respectively, a median sexual orientation disclosure index of 5/5 (i.e., disclosed to all categories outlined above) and 4/5 (i.e., had not disclosed to at least one of those categories, *P* < 0.001). Among those not recently tested, 68% had not disclosed their homosexuality to their father.

Recently tested MSM were also more likely to report having had casual male partners (87% versus 82%, *P* = 0.06) or steady male partners (82% versus 73%, *P* = 0.002) in the previous 6 months and to report ICU with male partners (62% versus 50%, *P* = 0.001) than their not recently tested counterparts. However, the total number of sexual male partners in the previous six months was not different between both MSM groups (overall median [IQR] = 11 [4–24]). Those recently tested tended to report less sex with women (10% versus 14%, *P* = 0.13) than those not recently tested. 

Both groups often reported sex under the influence of alcohol (62%). Those recently tested were more likely to report sex using poppers (46% versus 38%, *P* = 0.04) and, to a lesser extent, when they smoked cannabis (27% versus 22%, *P* = 0.18). However, they tended to report less sex under the influence of club drugs (9% versus 12%, *P* = 0.22) compared with not recently tested MSM. 

### 3.3. Factors Associated with Recent HIV Testing

After adjustment for the specific study, factors independently associated with recent HIV testing were as follows: the index of sexual orientation disclosure to relatives and friends (adjusted odds ratio, aOR = 1.2; 95% confidence interval, CI = [1.1–1.4]), ICU (aOR = 1.6; 95% CI = [1.2–2.1]), and sex under the influence of club drugs (aOR = 0.6, 95% CI = [0.4–1.0]) (Table 2). 

In the sensitivity analysis (Table 3), the model remained nearly unchanged when the 11 and 13 months cut-off points were used to define recent testing. 

## 4. Discussion

This is the first study investigating recent HIV testing among MSM living in France. Our analysis showed that approximately half of the study group (49%) had been tested for HIV in the 12 months previous to their participation in the CB-VCT programmes. These men were more likely to be out to all their relatives and friends and to report inconsistent condom use (ICU), but they were less likely to have had sex under the influence of club drugs when compared with those not tested for over one year or who had never been tested.

The two CB-VCT programmes reported here reached a significant proportion of MSM who had been recently tested for HIV as well as those who had not. However, our analysis was restricted to a convenience sample of the specific population of MSM who felt the need to be tested for HIV, so we did not provide information about men who were unwilling to be tested. It is important to underline that this study was based on MSM who decided to participate in an alternative HIV testing offer and who may have been reluctant to be tested using conventional HIV testing offers, or who experienced more discrimination based on the sexual orientation.

The French context regarding HIV testing has changed: the government recently decided to authorize the use of HIV rapid tests by nonmedical staff [30]. Currently, many HIV tests are performed by community members. It would be interesting to reconduct such study in a few months in order to verify whether the level of recent testing increased or not and whether the factors influencing the fact of being recently tested remain unchanged. 

The sensitivity analysis showed that using different cut-off points for the outcome variable (i.e., tested within the past 11, 12, and 13 months) did not drastically change the base-case model. Thus, the final multivariate model is stable and robust; the associations between the explanatory variables and the outcome are not exclusively due to the large size of our sample. 

Our results should be interpreted carefully as we cannot exclude recruitment bias arising from differences between the CB-VCT programmes in terms of study period and setting (*AIDES*' premises versus free and anonymous testing centres). However, the model was adjusted for the specific study in which men were enrolled, and no significant differences were found between both. In addition, the two programmes were carried out in large urban areas. Consequently, results cannot be extrapolated to MSM living in small towns or in the countryside, where living one's homosexuality openly is more complicated due to the fear of being recognized, the fear of outing and being labelled as gay and/or HIV positive.

The present study shows that half of the MSM involved had not been tested for HIV for more than one year. The current situation is far from adhering to French guidelines which recommend annual HIV testing for MSM [18]. Our rate of recent testing is comparable with those in many other studies among MSM conducted in resource-rich countries, from 43% in the UK to 54% in the USA [31–35]. However, one French study conducted among MSM attending gay venues in Paris (the Prevagay study) showed that 63% of participants had been tested within the previous 12 months [7]. These men reported attending various gay venues quite frequently, where HIV prevention is very present. It has been shown elsewhere that recent testing is associated with exposure to HIV prevention [36]; this may explain the higher rate of recently tested MSM in PREVAGAY compared with our study. Furthermore, attending such venues—identified as gay venues—requires the individual to be at least a little comfortable with his homosexuality in order to overcome the fear of outing. 

In our study, recently tested MSM had a higher index of sexual orientation disclosure (5/5) that is, they lived their homosexuality openly with all their relatives and friends, compared with nonrecently tested MSM (4/5). This finding has also been highlighted in a large US study among young MSM [33] and more recently in the European MSM Internet Survey (the EMIS study) [10, 37], where being out sexually to many people was positively associated with recent testing. In a recent French Internet survey on MSM, men who accessed—or who were interested in accessing—self-tests were also more likely to not have been tested recently and to live their sexual lives with men in absolute secrecy [38, 39]. 

Our results confirm that nondisclosure of sexual orientation is a major barrier to testing, and therefore to repeat testing among MSM. Nonrecently tested MSM were significantly less likely to be out; they were also more likely to define themselves as nonhomosexual and to report that they had sex with women compared with their recently tested counterparts. Interestingly, in a US study conducted among MSM, the desire to be perceived by others as heterosexual was negatively associated with recent testing, their belief being that “HIV testing is so gay” [35]. In Lebanon, a recent study highlighted that MSM who had disclosed their homosexuality to family and parents were more likely to have been tested for HIV [40]. In France, homosexuality is no longer criminalized and seems to be better accepted than in Lebanon for example, but many MSM do not live their homosexuality openly, probably because of quite widespread discrimination on the grounds of sexual orientation [41]. 

Unlike other studies which showed that a higher number of sex partners were associated with higher odds of recent testing [32, 35], we found no difference between recently and not recently tested MSM regarding the number of sex partners (high in both groups). On the other hand, those reporting ICU were significantly more likely to be recently tested. Not perceiving oneself to be at risk of acquiring HIV is a well-known barrier to HIV testing and is common in MSM as well as in the general population [23, 25, 42]. It is thus important to increase the self-perception of being at risk among those not recently tested and reporting ICU. 

The use of psychoactive products is associated with sexual risk behaviours and with a higher prevalence of HIV and other sexually transmitted diseases in MSM [43–46]. In our study, not recently tested MSM were significantly more likely to report sex under the influence of club drugs than their recently tested counterparts. The homosexuality disclosure variable seemed to play the role of what is known as a “suppressor variable” [47]: introducing the latter increased the explanatory significance of club drug use and consequently the quality of the model (i.e., log-likelihood (LL) was significantly higher when the homosexuality disclosure variable was introduced; LL_with  revelation  variable_ = −456.01 versus LL_without  revelation  variable_ = −474.05). This is not such a striking result in social science research, and particularly in behavioural studies [48]. The phenomenon suggests that club drugs use and homosexuality disclosure share important information and should be interpreted as a composite. Further qualitative research is needed to better identify behaviours, beliefs, and attitudes which mediate the relationship between club drug use, disclosure of homosexuality, and HIV testing.

Although repeating HIV testing is being promoted in MSM who engage in at-risk sexual behaviour, prevention efforts to reach MSM who are tested less frequently should be focused on those who use club drugs, particularly before sex, and those who live their homosexuality in relative secrecy. Increasing self-perception of risk among MSM should be an intervention included in a comprehensive and understandable prevention message for all MSM. In addition, governments should develop policies which encourage improved social acceptance of homosexuality, as concealment of sexual orientation represents a major barrier to testing and might impede health and well-being of sexual minorities.

## Figures and Tables

**Table 1:** Comparison of participants regarding the outcome: recently versus not recently tested (univariate analyses, *n* = 716).

Variables	Items	Whole sample (*n* = 716)	Recently tested (*n* = 349)	Not recently tested (*n* = 367)	*P* ∗
		*n* (%)	*n* (%)	*n* (%)	
Study enrolment	ANRS-DRAG	199 (27.8)	103 (29.5)	96 (26.2)	
	ANRS-COM'TEST	517 (70.5)	246 (70.5)	271 (73.8)	0.32
Demographics					
Age^§^	Median [IQR]	31 [25–39]	30 [25–38]	32 [25–40]	0.03
Education	<Secondary school certificate	93 (13)	44 (12.6)	49 (13.4)	
	≤2 years after secondary school	238 (33.2)	119 (34.1)	119 (32.4)	0.66
	>2 years after secondary school	374 (52.2)	179 (51.3)	195 (53.1)	0.92
Being in active employment	No	142 (19.8)	72 (20.6)	70 (19.1)	
	Yes	563 (78.6)	272 (77.9)	291 (79.3)	0.61
Being single	No	208 (29.1)	94 (26.9)	114 (31.1)	
	Yes	506 (70.7)	255 (73.1)	251 (68.4)	0.21
Sexual orientation, disclosure, and victim of verbal abuse or aggression					
Sexual orientation	Homosexual	582 (81.3)	299 (85.7)	283 (77.1)	
	Bisexual	94 (13.1)	38 (10.9)	56 (15.3)	0.05
	Heterosexual	9 (1.3)	0 (0)	9 (2.5)	na
	Other	27 (3.8)	10 (2.9)	17 (4.6)	0.15
Index of sexual orientation disclosure^§^	Median [IQR]	4 [2–5]	5 [3–5]	4 [1–5]	<0.001
Victim of verbal abuse because of sexual orientation	No	570 (79.6)	263 (75.4)	307 (83.7)	
	Yes	143 (20.0)	85 (24.4)	58 (15.8)	0.005
Victim of aggression because of sexual orientation	No	682 (95.3)	331 (94.8)	351 (95.6)	
	Yes	22 (3.1)	11 (3.2)	11 (3.0)	0.89
Sexual life (previous 6 months)					
Total no. of sex male partners^§^	Median [IQR]	11 [4–24]	11 [5–30]	10 [3–22]	0.48
Having casual male partners	No	111 (15.5)	45 (12.9)	66 (18.0)	
	Yes	605 (84.5)	304 (87.1)	301 (82.0)	0.06
No. of casual male partners^§^	Median [IQR]	8 [3–20]	10 [3–22]	7 [2–20]	0.53
Having steady male partners	No	163 (22.7)	62 (17.8)	101 (27.5)	
	Yes	553 (77.2)	287 (82.2)	266 (72.5)	0.002
No. of steady male partner(s)^§^	Median [IQR]	2 [1–4]	2 [1–4]	2 [0–4]	0.58
ICU	No	293 (40.9)	121 (34.7)	172 (46.9)	
	Yes	401 (56.0)	216 (61.9)	185 (50.4)	0.001
Sex with women	No	592 (82.7)	297 (85.1)	295 (80.4)	
	Yes	87 (12.2)	36 (10.3)	51 (13.9)	0.13
Sex under the influence of drugs (previous 6 months)					
Alcohol	No	275 (38.4)	127 (36.4)	148 (40.3)	
	Yes	441 (61.6)	222 (63.6)	219 (59.7)	0.28
Poppers	No	418 (58.4)	190 (54.4)	228 (62.1)	
	Yes	298 (41.6)	159 (45.6)	139 (37.9)	0.04
Cannabis	No	541 (75.6)	256 (73.4)	285 (77.7)	
	Yes	175 (24.4)	93 (26.6)	82 (22.3)	0.18
Club drugs∗∗	No	640 (89.4)	317 (90.8)	323 (88.0)	
	Yes	76 (10.6)	32 (9.2)	44 (12.0)	0.22

^*^This column displays *P* values for each variable tested in univariate logistic regression, showing whether differences between recently tested and not recently tested MSM are significant or not; ^**^Ecstasy, MDMA, cocaine, or crack.

^§^Medians were used because these variables did not follow a normal distribution.

IQR: interquartile range; ICU: inconsistent condom use with casual and/or steady male partners; na: not applicable.

**Table 2:** Factors independently associated with recent testing, adjusted for the study (multivariate analysis, *n* = 685^*^).

Variables	Items	Recently tested % (*n* = 336)	Not recently tested % (*n* = 349)	OR [95% CI]	*P *	aOR [95% CI]	*P *
Index of sexual orientation disclosure^§^	Median [IQR]	5 [3–5]	4 [1–5]	1.2 [1.1–1.4]	<0.001	1.2 [1.1–1.4]	<0.001
ICU	No	36	48	1		1	
	Yes	64	52	1.7 [1.2–2.3]	0.001	1.6 [1.2–2.1]	0.005
Club drugs∗∗	No	91	87	1		1	
	Yes	10	13	0.7 [0.5–1.2]	0.22	0.6 [0.4–1.0]	0.05
Study enrolment	ANRS-COM'TEST	70	74	1		1	
	ANRS DRAG	30	26	1.2 [0.9–1.6]	0.32	1.1 [0.8–1.6]	0.43

^*^Valid dataset for all variables of the model; ^**^Ecstasy, MDMA, cocaine, or crack.

^§^Median was used because this variable did not follow a normal distribution.

OR: odds ratio; aOR: adjusted odds ratio; CI: confidence interval; IQR: interquartile range; ICU: inconsistent condom use with casual and/or steady male partners. Log-likelihood = −456.01.

**Table 3:** Sensitivity analysis: factors independently associated with recent testing, adjusted for the study and using different cut-off points for the outcome (multivariate analysis, *n* = 685^*^).

(Recently tested/not recently tested)		12-month cutoff (base-case analysis) (49%/51%)		11-month cutoff (46%/54%)		13-month cutoff (51%/49%)	
Variables	Items	aOR [95% CI]	*P *	aOR [95% CI]	*P *	aOR [95% CI]	*P *
Index of sexual orientation disclosure^§^	Median [IQR]	1.2 [1.1–1.4]	<0.001	1.2 [1.1–1.3]	<0.001	1.2 [1.1–1.3]	<0.001
ICU	No	1		1		1	
	Yes	1.6 [1.2–2.1]	0.005	1.7 [1.2–2.3]	0.001	1.5 [1.1–2.1]	0.008
Club drugs∗∗	No	1		1		1	
	Yes	0.6 [0.4–1.0]	0.05	0.6 [0.4–1.0]	0.05	0.6 [0.4–1.0]	0.05
Study enrolment	ANRS-COM'TEST	1		1		1	
	ANRS DRAG	1.1 [0.8–1.6]	0.43	1.3 [0.9–1.9]	0.12	1.17 [0.8–1.7]	0.35

^*^Valid dataset for all variables of the model; ^**^Ecstasy, MDMA, cocaine, or crack.

^§^Median was used because this variable did not follow a normal distribution.

aOR: adjusted odds ratio; CI: confidence interval; IQR: interquartile range; ICU: inconsistent condom use with casual and/or steady male partners.
